# Systematic Lymphadenectomy and Oncological Outcomes of Women With Apparent Early-Stage Clear Cell Carcinoma of the Endometrium: A Multi-Institutional Cohort Study

**DOI:** 10.3389/fonc.2022.800957

**Published:** 2022-03-24

**Authors:** Yong Tian, Lin Ran, Yi Liu, Yu Xu, Juan Shen, Gong-sheng Mi, Feng-mei Ke

**Affiliations:** ^1^ Department of Obstetrics and Gynecology, Central Hospital of Enshi Tujia and Miao Autonomous Prefecture, Enshi Clinical College of Wuhan University, Enshi, China; ^2^ Department of Obstetrics and Gynecology, West China Second University Hospital, Chengdu, Sichuan University, Chengdu, China; ^3^ Department of Obstetrics and Gynecology, Mianyang Central Hospital, Mianyang, China

**Keywords:** lymphadenectomy, clear cell carcinoma, endometrial cancer, overall survival, disease-free survival

## Abstract

**Objective:**

The survival value of systematic lymphadenectomy for endometrial cancer is ambiguous and controversial. The current study aimed to evaluate the long-term survival role of combined pelvic and para-aortic lymphadenectomy in patients with presumed early-stage clear cell carcinoma of the endometrium.

**Methods:**

Patients in three Chinese teaching hospitals who presented between 2012 and 2017 with apparent early-stage clear cell carcinoma of the endometrium and underwent surgical staging were selected. Patients who did and did not undergo systematic lymphadenectomy were identified and clinicopathological characteristics were compared. Disease-free survival and overall survival were evaluated following the generation of the Kaplan-Meier curves and the comparison using the log-rank test. A Cox proportional hazards model was employed to control for confounders.

**Results:**

A total of 244 patients underwent systematic lymphadenectomy and 89 did not receive lymph node dissection. The demographic and baseline data were comparable between the two groups. The rate of disease-free survival at 5 years was 64.10% in patients who underwent systematic lymphadenectomy and 45.05% in patients who did not undergo lymphadenectomy. Patients who underwent systematic lymphadenectomy had better disease-free survival than those who did not receive lymphadenectomy (HR, 0.54. 95% CI, 0.38-0.76. *P*=0.000). The rate of 5-year overall survival was 68.87% in the lymphadenectomy group and 53.33% in patients who did not undergo systematic lymphadenectomy. Systematic lymphadenectomy was also associated with improved 5-year overall survival for women with presumed early-stage clear cell carcinoma of the endometrium (HR, 0.58. 95% CI, 0.39-0.85. *P*=0.005). After adjusting for confounders, systematic lymphadenectomy was still independently associated with improved disease-free survival and overall survival.

**Conclusion:**

Patients with apparent early-stage clear cell carcinoma of the endometrium who underwent systematic lymphadenectomy had better long-term survival than those who did not undergo systematic lymphadenectomy.

## Introduction

Endometrial cancer is the most common malignancy of the female reproductive system in developed countries (1–4). Close to three-quarters of patients with endometrial cancer have an early-stage disease, and 5-year overall survival rates exceed 90% (1, 2). Clear cell carcinoma of the endometrium is a rare subtype of endometrial cancer, accounting for less than 6% of all endometrial cancer cases (5, 6). Compared with endometrioid endometrial cancer, clear cell carcinoma of the endometrium is considered to be more aggressive, has a higher risk of recurrence, and has a worse prognosis (7, 8).

For endometrial cancer, surgical staging is the mainstay of the initial management, which includes at least total extrafascial hysterectomy and bilateral salpingo-oophorectomy (9). Staging is based on pathological evaluation of the specimen and can be employed to stratify the prognosis and identify women who may benefit from postoperative adjuvant therapy.

Surgical staging for women with endometrial cancer has historically included regional lymph node resection. The National Comprehensive Cancer Network (NCCN) recommends “lymph node assessment” for apparent early-stage cases, a term that includes sentinel lymph node mapping and systematic lymphadenectomy, and reflects the heterogeneity of clinical practice and controversy regarding the extent and approach to lymphadenectomy in the management of endometrial cancer (10). For low-risk early-stage endometrial cancer, two large prospective trials have failed to show a survival benefit associated with systematic lymphadenectomy (11, 12). In terms of high-risk endometrial cancer, the currently popular practice is to perform combined pelvic and para-aortic lymphadenectomy (13). However, this practice was mainly based on evidence from retrospective studies (13, 14). What is more, due to the rarity of clear cell carcinoma of the endometrium, the proportion of clear cell carcinoma of the endometrium in these studies was very low (13, 14). Therefore, the long-term survival value of systematic lymphadenectomy for apparent early-stage clear cell carcinoma of the endometrium is still unclear.

Taken together, we conducted this multi-institutional cohort study to explore the ontological effect of systematic lymphadenectomy on women with apparent early-stage clear cell carcinoma of the endometrium.

## Materials and Methods

### Study Design and Population

This was a multicenter retrospective cohort study involving three Chinese tertiary teaching hospitals. In consideration of the retrospective nature of this study and this research did not involve any identifiable private information, the ethics review and informed consent to participate were exempted by the Institutional Review Boards of the participating hospitals. This research was conducted following the Declaration of Helsinki (15).

The medical record systems of these participating hospitals were queried, and a cohort of women diagnosed with endometrial cancer between January 2012 and December 2017 was identified. Based on the International Classification of Disease-O-3 histology codes, patients who were diagnosed with a histologically confirmed clear cell carcinoma were identified. Patients were included in this study if they met the following inclusion criteria: (1) undergoing at least a total hysterectomy and bilateral salpingo-oophorectomy, (2) physical examination and the preoperative imaging examinations (pelvic ultrasound, computerized tomography, or magnetic resonance imaging) did not find any signs of advanced disease such as vaginal involvement, extrauterine metastases, or enlarged regional lymph nodes, (3) did not undergo any neoadjuvant therapy, and (4) undergoing consecutive follow-up at these hospitals. Patients were excluded from this study if they had synchronous malignancy, were pregnant, had a history of other cancers, only underwent pelvic lymphadenectomy, or were in a state of immunosuppression.

All patients included in this study were staged according to the 2009 International Federation of Gynecology and Obstetrics (FIGO) staging system based on the pathological examination of specimens. After initiative treatment, all patients were suggested to undergo an outpatient follow-up visit every three months within the first two years, every six months in the third year, and once a year thereafter. At each postoperative follow-up visit, the patients underwent at least a pelvic exam, a Pap test, a carbohydrate antigen 199 and carbohydrate antigen 125 blood test, and a pelvic ultrasound. Other imaging tests or biopsies would be done to identify disease recurrence based on the physical exam, laboratory tests, and any changes the patient reports.

### Data Collection

Data on the following demographic, clinicopathological, and treatment variables were extracted from the electronic medical records of the eligible patients: year of diagnosis, age at diagnosis, marital status at diagnosis, preoperative American Society of Anesthesiologists (ASA) physical status score, body mass index (BMI) at diagnosis, postoperative disease staging results (2009 FIGO staging system), tumor size, results of peritoneal cytology, the status of lymph-vascular space invasion (LVSI), approach of surgical staging (laparoscopy or laparotomy), scope of surgical staging (systematic lymphadenectomy, the number of regional lymph nodes removed, and omentectomy), and protocol of adjuvant therapy (chemotherapy, radiotherapy, or chemoradiotherapy). In the current study, systematic lymphadenectomy was defined as combined pelvic and para-aortic lymphadenectomy. Radiotherapy included vaginal brachytherapy and/or external beam radiotherapy. Any patient who received chemotherapy and/or radiotherapy within the first six months after surgical staging was considered to have received adjuvant therapy.

The endpoints of follow-up for this study were all-cause death or January 1, 2020. The survival data collected were as follows: vital status, time of death, time of disease recurrence, and site of disease recurrence.

### Outcomes of Interest

Outcomes of interest in this study were disease-free survival (DFS) and overall survival (OS). DFS was defined as the date from the surgery for endometrial cancer to the date of disease recurrence, last follow-up, or death from any cause. OS was defined as the duration of time from the start of surgery for endometrial cancer to last follow-up or death from any cause.

### Statistical Analysis

Included patients were divided into two groups according to whether they have undergone systematic lymphadenectomy. The demographic, clinicopathological, and treatment variables were compared between the two groups. The frequency of distribution of categorical variables was compared using the Chi-square test. Continuous variables were compared using a *t*-test for independent samples to test the equality of means or using the Wilcoxon rank-sum test to compare medians for non-normally distributed variables. The Kaplan–Meier curves were drawn to determine the 5-year DFS rate and the 5-year OS rate. DFS and OS were compared using the log-rank test. The Cox proportional hazards regression was employed to determine the hazard ratios (HRs), adjusted hazard ratios (aHRs), and 95% confidence intervals (CIs) of each variable. Multivariate analyses were performed to adjust for confounding factors. Variables with a *P* value of less than 0.2 in the univariate analysis were considered as confounding factors and were included in the Cox proportional hazards regression model.

All statistical analyses were conducted using the Statistical Package for the Social Sciences (SPSS, v.25) statistical package (International Business Machines Corporation Armonk, New York). The Kaplan–Meier curves in this study were generated by STATA (v15; STATA, College Station, TX; Computing Resource Center, Santa Monica, CA). The level of statistical significance in this study was set at 0.05.

## Results

### Characteristics of the Study Cohort

**Figure 1:**
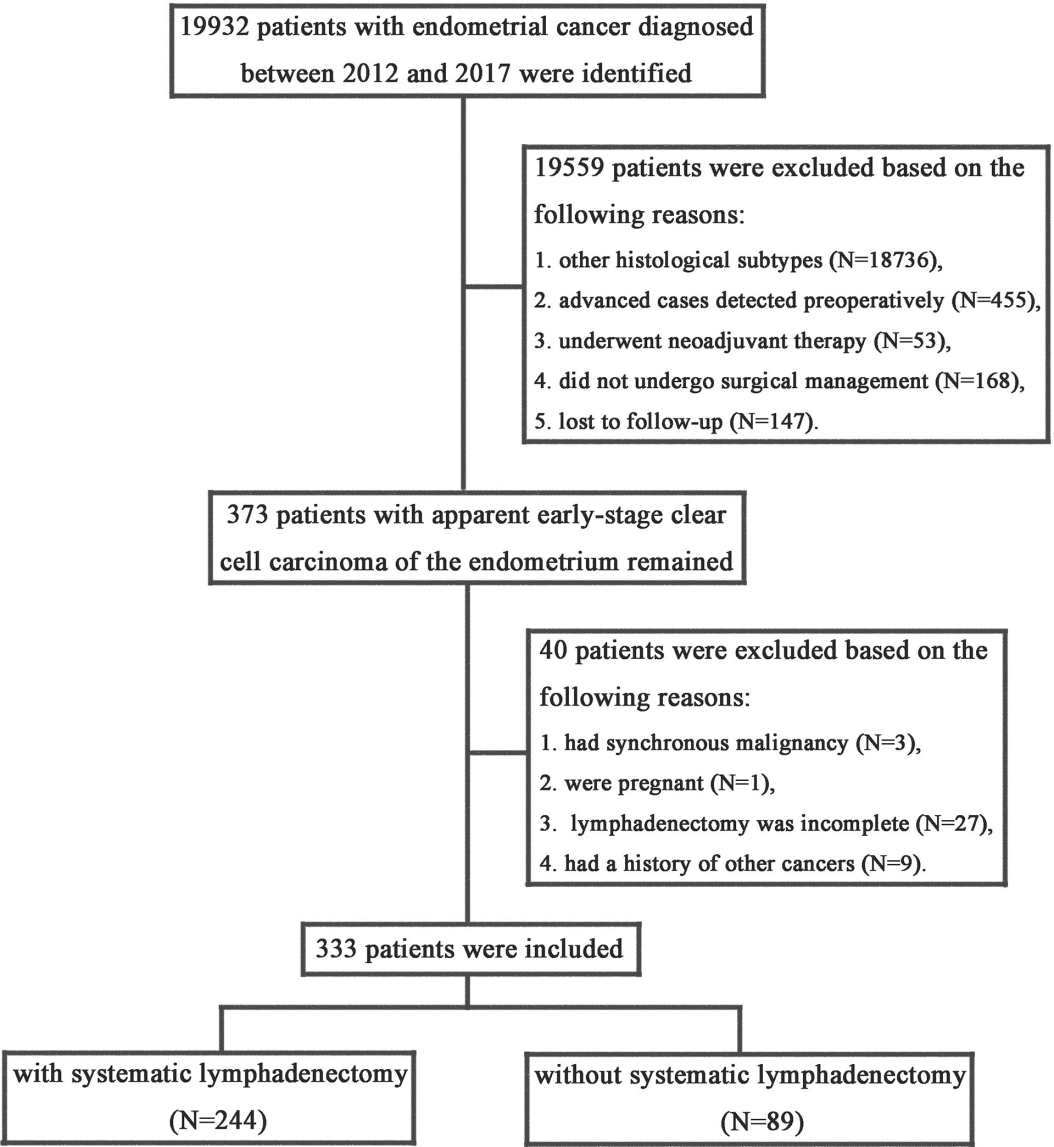
Flow chart of patients selection.

A total of 19932 women were diagnosed with endometrial cancer between January 2012 and December 2017 at the three participating hospitals. After excluding cases that did not meet the inclusion criteria, a total of 333 patients were included in this study. **Figure 1** shows the process of the case selection.

Eligible patients were divided into two groups based on whether or not they underwent systematic lymphadenectomy. Of the included patients, 244 patients underwent combined pelvic and para-aortic lymphadenectomy and the remaining 89 patients did not undergo regional lymph node removal. The baseline characteristics of the study cohort are summarized in **Table 1**. The treatment groups were balanced with respect to baseline characteristics. The mean age of the overall cohort at diagnosis was 66.8 years (standard deviation, 9.06). The median follow-up time was 49 months (range, 4-107) for the patients who underwent systematic lymphadenectomy and was 48 months (range, 1-105) for patients who were with regional lymph nodes reserved. The distribution of patients by preoperative ASA physical status score (*P*=0.913) and BMI (*P*=0.807) was also similar in the two groups.

**Table 1:** Demographics and Baseline Characteristics of the study cohort.

	Total (N=333)	With lymphadenectomy (N=244)	Without lymphadenectomy (N=89)	*P*
Year of diagnosis				0.131
2012-2014	140 (42.0%)	99 (40.6%)	41 (46.1%)	
2015-2017	193 (58.0%)	145 (59.4%)	48 (53.9%)	
Age at diagnosis	66.8 ± 9.06	66.4 ± 8.80	68.0 ± 9.68	0.675
Marital status				0.759
Married	197 (59.2%)	146 (59.8%)	51 (57.3%)	
Single	61 (18.3%)	46 (18.9%)	15 (16.9%)	
Unknown	66 (22.5%)	52 (21.3%)	23 (25.8%)	
ASA^1^ physical status score				0.913
I	72 (21.6%)	51 (20.9%)	21 (23.6%)	
II	133 (39.9%)	96 (39.3%)	37 (41.6%)	
III	84 (25.2%)	63 (25.8%)	21 (23.6%)	
IV	44 (13.2%)	34 (13.9%)	10 (11.2%)	
BMI^2^ (Kg/m^2^)	22.5 ± 3.51	22.6 ± 3.73	22.1 ± 3.25	0.807
Duration of follow-up (month)	46.0 (1.00, 107)	49.0 (4-107)	48.0 (1.00-105)	0.914
Stage (FIGO^3^ 2009)				N/A^4^
IA	79 (23.7%)	60 (24.6%)	19 (21.3%)	
IB	60 (18.0%)	45 (18.4%)	15 (16.9%)	
II	42 (12.6%)	29 (11.9%)	13 (14.6%)	
IIIA	40 (12.0%)	32 (13.1%)	8 (9.0%)	
IIIC	83 (24.9%)	74 (30.3%)	9 (10.1%)	
IV	12 (3.6%)	4 (1.6%)	8 (9.0%)	
Not reported	17 (5.1%)	0 (0.0%)	17 (19.1%)	
Tumor size				0.755
< 2cm	169 (50.8%)	127 (52.0%)	42 (47.2%)	
2cm - 4cm	113 (33.9%)	36 (14.8%)	15 (16.9%)	
> 4cm	51 (15.3%)	81 (33.2%)	32 (36.0%)	
Peritoneal cytology				> 0.999
Negative	295 (88.6%)	215 (88.1%)	80 (89.9%)	
Positive	38 (11.4%)	29 (11.9%)	9 (10.1%)	
lymph-vascular space invasion				> 0.999
No	255 (76.6%)	185 (75.8%)	70 (78.7%)	
Yes	78 (23.4%)	59 (24.2%)	19 (21.3%)	
Surgical approach				0.021
Laparoscopic surgery	218 (65.5%)	168 (68.9%)	50 (56.2%)	
Open	115 (34.5%)	76 (31.1%)	39 (43.8%)	
Omentectomy				
No	181 (54.4%)	135 (55.3%)	46 (51.7%)	0.303
Yes	152 (45.6%)	109 (44.7%)	43 (48.3%)	
Adjuvant therapy				0.103
No	54 (16.2%)	37 (15.2%)	17 (19.1%)	
Chemotherapy	113 (33.9%)	82 (33.6%)	31 (34.8%)	
Radiotherapy	45 (13.5%)	30 (12.3%)	5 (16.9%)	
Chemoradiotherapy	121 (36.3%)	95 (38.9%)	26 (29.2%)	

^1^American Society of Anaesthesiologists.

^2^Body Mass Index.

^3^The International Federation of Gynecology and Obstetrics.

^4^Not Applicable.

There were no significant differences between the two groups with respect to the proportion of tumor size of 2 cm or greater (48.0% and 52.9%, *P*=0.755), LVSI (24.2% and 21.3%, *P* > 0.999), and positive peritoneal cytology (11.9% and 10.1%, *P* > 0.999).

In terms of the approach of surgical staging, the rate of laparoscopic surgery in the systematic lymphadenectomy group was 68.9%, while the rate in the nodes reserved group was 56.2%. In the entire study cohort, more than half of the patients (54.4%) underwent omentectomy, 40.5% of the patients with presumed early-stage cases were classified as advanced stages (FIGO stage III or IV) postoperatively. Rates of postoperative adjuvant therapy were similar in the two groups, chemoradiotherapy (36.3%) was the most common adjuvant treatment modality, followed by chemotherapy (33.9%).

### Survival Outcomes

At the time of the analysis, 56 patients had a disease recurrence, 27 in the cohort without systematic lymphadenectomy and 29 in the cohort with systematic lymphadenectomy. In patients without systematic lymphadenectomy, the most recurrence was nodal recurrences (15.7%), followed by multi-site recurrence (4.5%). While among patients who underwent systemic lymphadenectomy, the most frequent recurrence site was the pelvis (2.5%) and abdomen (2.5%). There were significant differences in disease recurrence rates (*P*=0.013) and recurrence patterns (*P*=0.000) between the two groups of patients. **Table 2** shows the patterns and rates of disease recurrence in our study cohort. A total of 115 deaths were observed during the follow-up period, 40 in the cohort without systematic lymphadenectomy and 75 in the cohort with systematic lymphadenectomy.

**Table 2:** Patterns and rates of recurrence by systematic lymphadenectomy vs. nodes conserved.

	Without lymphadenectomy	With lymphadenectomy	*P*
	(N=89)	(N=244)	
Recurrence			0.013
No	62 (69.7%)	215 (88.1%)	
Yes	27 (30.3%)	29 (11.9%)	
Site of recurrence			0.000
Vagina	1 (1.1%)	4 (1.6%)	
Pelvis	3 (3.4%)	6 (2.5%)	
Abdomen	2 (2.2%)	6 (2.5%)	
Nodal	14 (15.7%)	3 (1.2%)	
Liver	0 (0.0%)	4 (1.6%)	
Lung	1 (1.1%)	3 (1.2%)	
Bone	2 (2.2%)	1 (0.4%)	
Multiple	4 (4.5%)	2 (0.8%)	

**Figure 2:**
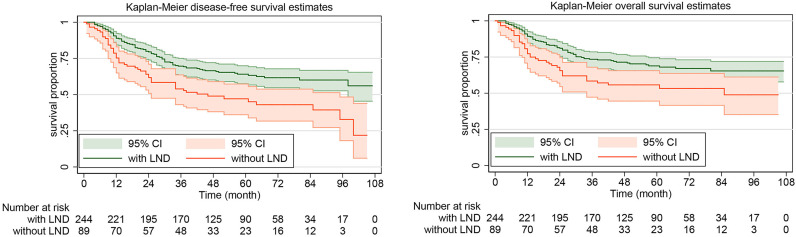
Kaplan-Meier curves of disease-free survival and overall survival for patients with apparent early-stage clear cell carcinoma of the endometrium, by whether or not systematic lymphadenectomy was performed. LND, lymph node dissection.

**Figure 3:**
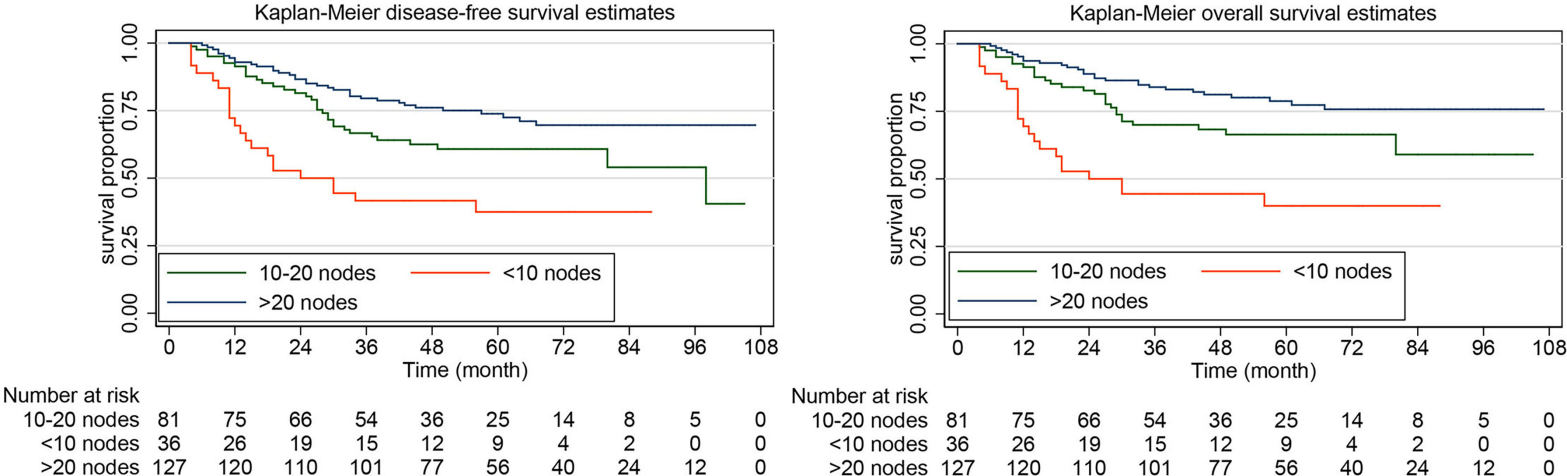
Kaplan-Meier curves of disease-free survival and overall survival for patients with apparent early-stage clear cell carcinoma of the endometrium, by the number of the lymph nodes removed.

The rate of DFS at 5 years was 64.10% (95% CI, 57.53%-69.92%) in the systematic lymphadenectomy group and 45.05% (95% CI, 33.88%-55.58%) among the patients who did not undergo systematic lymphadenectomy (**Figure 2**). The survival analysis by log-rank test showed that systematic lymphadenectomy was significantly associated with better DFS (HR, 0.54. 95% CI, 0.38-0.76. *P*=0.000). Compared with patients who underwent systematic lymphadenectomy, patients who did not undergo lymph node resection had a lower 5-year survival rate. Their five-year overall survival rates were 68.87% (95% CI, 62.33%-74.51%) and 53.33% (95% CI, 41.63%-63.69%), respectively. The log-rank test indicated that systematic lymphadenectomy was significantly associated with better OS (HR, 0.58. 95% CI, 0.39-0.85. *P*=0.005). Among patients who underwent systematic lymphadenectomy, the survival analyses were also investigated according to subclassification of the number of lymph nodes removed. Compared with patients who had less than ten regional lymph nodes removed, patients with ten to twenty or more than twenty lymph nodes removed experienced better DFS and OS (**Figure 3**).

### Multivariate Analysis

The Cox proportional hazards regression model was employed for additional study of the superiority of systematic lymphadenectomy for patients with apparent early-stage clear cell carcinoma of the endometrium to control for the potential confounding factors. Based on the results of univariate analysis (**Supplementary Material 1**), the potential confounding factors that were included in the Cox proportional hazards regression model were as follows: age at diagnosis, marital status, preoperative ASA physical status score, stage, tumor size, nodal involvement, surgical approach, and postoperative adjuvant therapy. After the analysis was adjusted for these variables, systematic lymphadenectomy was still independently associated with improved DFS (aHR, 0.57. 95% CI, 0.39-0.85. *P*=0.005) and OS (aHR, 0.64. 95% CI, 0.41-0.99. *P*=0.047) in patients with apparent early-stage clear cell carcinoma of the endometrium (**Table 3**).

**Table 3:** Multivariate analyses of prognostic factor for DFS and OS in women with apparent early-stage clear cell carcinoma of the endometrium.

	DFS^1^			OS^2^		
	aHR^3^	95% CI^4^	*P*	aHR	95% CI	*P*
Age at diagnosis						
< 65 years	Reference			Reference		
> 65 years	1.42	0.96-2.10	0.078	1.31	0.75-1.71	0.556
Marital status						
Married	Reference			Reference		
Single	2.10	0.99-4.41	0.051	1.88	0.85-5.16	0.122
Unknown	1.33	0.83-2.14	0.231	1.30	0.79-2.14	0.299
ASA^5^ physical status score						
I/II	Reference			Reference		
III/IV	1.50	1.03-2.37	0.007	1.60	1.20-3.54	0.037
Stage (FIGO^6^ 2009)						
I/II	Reference			Reference		
III/IV	4.75	2.68-8.43	0.000	4.90	2.59-9.27	0.000
Tumor size						
< 2cm	Reference			Reference		
2cm - 4cm	1.01	0.68-1.49	0.961	1.18	0.77-1.80	0.452
> 4cm	1.79	1.15-4.31	0.010	1.74	1.03-4.81	0.021
Nodal involvement						
No	Reference			Reference		
Yes	2.13	1.09-4.14	0.026	2.03	1.03-4.39	0.041
Surgical approach						
Open	Reference					
Laparoscopic surgery	1.53	0.75-3.12	0.246			
Systematic lymphadenectomy						
No	Reference			Reference		
Yes	0.57	0.38-0.85	0.005	0.64	0.41-0.99	0.047
Adjuvant therapy						
No	Reference			Reference		
Chemotherapy	0.67	0.40-0.96	0.002	0.63	0.42-0.95	0.028
Radiotherapy	0.76	0.34-0.94	0.016	0.71	0.39-0.90	0.009
Chemoradiotherapy	0.58	0.35-0.87	0.002	0.56	0.25-0.87	0.021

^1^Disease-free Survival.

^2^Overall Survival.

^3^Adjusted Hazard Ratio.

^4^Confidence Interval.

^5^American Society of Anaesthesiologists.

^6^The International Federation of Gynecology and Obstetrics.

Moreover, the results of the multivariate analysis also revealed that preoperative ASA physical status score, postoperative disease FIGO stage, tumor size, nodal involvement, and postoperative adjuvant therapy were independent predictors for survival in patients with apparent early-stage clear cell carcinoma of the endometrium.

## Discussion

In this multi-institutional retrospective cohort study, patients who underwent systematic lymphadenectomy for apparent early-stage clear cell carcinoma of the endometrium had better long-term survival and a lower risk of disease recurrence than patients who did not undergo systematic lymphadenectomy. Our results justify the employment of combined pelvic and para-aortic lymphadenectomy for patients with apparent early-stage clear cell carcinoma of the endometrium.

The practice of systematic lymphadenectomy for early-stage endometrial cancer has been controversial. The role of regional lymph nodes resection for endometrial cancer was established based on the results of a landmark Gynecologic Oncology Group (GOG) study, GOC 33, in 1987 (16). This study included patients with apparent early-stage disease and identified that the risk of regional lymph node metastasis was correlated with the degree of tumor differentiation and the depth of myometrial invasion (16). Since then, systematic lymphadenectomy has become a part of the surgical staging for endometrial cancer.

Previously, some retrospective studies have suggested that lymph node resection has a therapeutic effect on endometrial cancer (14, 17–19). This therapeutic effect was especially significant for patients with intermediate-risk and high-risk endometrial cancer (14, 17–19). Cragun et al. found that women with clinical early-stage high-risk endometrial cancer having more than 11 regional lymph nodes removed had improved OS (HR, 0.25. P <.0001) and progression-free survival (HR, 0.26. P <.0001) compared with women having 11 or fewer nodes removed (17). The data from the Surveillance, Epidemiology and End Results Program between 1988-2001 involving 12333 patients also demonstrated that a more extensive lymph node resection was associated with improved 5-year disease-specific survival in high-risk endometrial cancer (19).

However, the results of two large prospective clinical studies did not indicate a survival benefit associated with lymph nodes resection (11, 12). The UK Medical Research Council-A Study in the Treatment of Endometrial Cancer (MRC-ASTEC) trial, with 1408 women of clinical early-stage endometrial cancer included, reported that systematic lymphadenectomy failed to improve the overall survival (aHR, 1.04. 95% CI, 0.74-1.45. *P*=0.83) and recurrence-free survival (aHR, 1.25. 95% CI, 0.93-1.66. *P*=0.14) of the study cohort (11). Another randomized controlled trial also explored the survival effect of lymphadenectomy on clinical early-stage endometrial cancer. This study found that although systematic lymphadenectomy statistically significantly improved surgical staging, it did not improve DFS and OS (12). A systematic review and meta-analysis conducted in 2017 also found no evidence that systematic lymphadenectomy decreases the likelihood of death or disease recurrence compared with no lymphadenectomy in women with presumed early-stage endometrial cancer (20). What is more, this meta-analysis found that systematic lymphadenectomy was associated with an increased risk of surgery-related systemic morbidity or lymphoedema formation (20).

Clear cell carcinoma of the endometrium, as a rare yet aggressive subtype of endometrial cancer, presents a worse prognosis when compared with endometrioid carcinoma. The reported 5-year OS rate for the advanced disease was 42.3% to 62.5%, and hence more extensive scope of surgical staging and aggressive adjuvant therapy is favored (21–23). Among patients with no evidence of endometrial stromal or myometrial invasion, there is still a considered high risk of regional lymph node involvement. A multi-institutional cohort study conducted by Abdulfatah et al. reported that up to 30% (40/135) of patients with clear cell carcinoma of the endometrium have positive lymph nodes (24). This result is consistent with the findings of our research. In the current study, 244 patients who underwent systematic lymphadenectomy, positive lymph nodes were documented in 74 cases. Systematic lymphadenectomy has two effects for this kind of disease with a high risk of lymph node involvement. On the one hand, lymphadenectomy can remove the draining lymph tissue from the area where cancer has metastasized, reduce the risk of local or distant recurrence of the disease, and be therapeutic. The therapeutic role of lymph node resection for endometrial cancer has been confirmed by many studies (14, 17–19). On the other hand, lymphadenectomy can identify the risk factors for death and disease recurrence, individualize the following adjuvant therapy, and be diagnostic. Using the data from the Surveillance, Epidemiology, and End Results Program, Matsuo et al. carried out a study to explore the association between the extent of lymphadenectomy and the use of adjuvant radiotherapy for early-stage endometrial cancer (25). They found that surgeons and radiation oncologists tended to evaluate the extent of lymphadenectomy when counseling women with early-stage endometrial cancer for postoperative radiation (25).

Systematic lymphadenectomy can also have some negative effects on patients with endometrial cancer. The common long-term postoperative complications associated with systematic lymphadenectomy include lymphedema and lymphocele (26–28). The data from a high-volume center indicated that, after a mean follow-up duration of 53.2 months, the incidence of postoperative lower limb lymphedema and lymphocele in patients with endometrial cancer having systematic lymphadenectomy was 36.9% and 17.3%, respectively (26). Hence, sentinel lymph node mapping is becoming a promising option for apparent early-stage low-risk endometrial cancer (29, 30). For high-grade endometrial cancer, sentinel lymph node mapping also seems to be a viable option for surgical staging (31). However, we need more high-quality studies to confirm the safety and feasibility of sentinel lymph node mapping for high-risk endometrial cancer (clear cell carcinoma included).

For aggressive histotypes of gynecologic malignancies (clear cell carcinoma of the endometrium included), we believe there are other research topics of great clinical significance. For example, the long-term oncological safety of minimally invasive surgery (MIS) for women with these aggressive tumors. The study of Gallotta et al. has demonstrated that for appropriately selected women with aggressive gynecologic cancer, MIS is safe and feasible (32). However, randomized controlled trials are still needed. Another research topic that deserves our attention is whether to treat recurrent gynecological tumors (nodal recurrence included) with salvage surgery or chemoradiotherapy. This topic is extremely controversial and of high importance, some leading researchers have done some studies on it (33). However, more research is needed to reach a consensus.

Using data from three high-volume centers offering a diverse patient population, our study included a relatively large sample size and specifically evaluated the survival value of systematic lymphadenectomy for patients with presumed early-stage clear cell carcinoma of the endometrium. Most of the included patients underwent comprehensive surgical staging and were followed up for a relatively long period. However, some limitations still exist in our study. First, there is some inherent bias such as recall bias and selection bias because of the retrospective nature of this current study. Second, when using electronic medical records to identify potential qualified patients, potential information bias is also a concern. Last, we did not conduct a second pathological review by a pathologist who specializes in gynecological malignancies due to the limited resources.

## Conclusion

In conclusion, systematic lymphadenectomy can improve the long-term survival of patients with presumed early-stage clear cell carcinoma of the endometrium. Ideally, prospective clinical trials shall provide insight into the most effective surgical management modalities.

## Data Availability Statement

The data sets used and/or analyzed during the current study are available from the corresponding author on reasonable request.

## Ethics Statement

Ethical review and approval was not required for the study on human participants in accordance with the local legislation and institutional requirements. Written informed consent for participation was not required for this study in accordance with the national legislation and the institutional requirements.

## Author Contributions

Conceptualization: YT, LR, and YX. Methodology: YT, LR, F-mK, and YX. Data collection: all authors. Project administration: YX and F-mK. Supervision: F-mK. Writing - original draft: YT, LR, and YX. Writing - review and editing: all authors. All authors contributed to the article and approved the submitted version.

## Conflict of Interest

The authors declare that the research was conducted in the absence of any commercial or financial relationships that could be construed as a potential conflict of interest.

## Publisher’s Note

All claims expressed in this article are solely those of the authors and do not necessarily represent those of their affiliated organizations, or those of the publisher, the editors and the reviewers. Any product that may be evaluated in this article, or claim that may be made by its manufacturer, is not guaranteed or endorsed by the publisher.
