# Supplementary material for: Systematic Lymphadenectomy and Oncological Outcomes of Women With Apparent Early-Stage Clear Cell Carcinoma of the Endometrium: A Multi-Institutional Cohort Study
Source: Front Oncol. 2022 Mar 24;12:800957. doi: 10.3389/fonc.2022.800957 (PMC8987355; doi:10.3389/fonc.2022.800957)
Supplement: Supplementary file 1 [file Table_1.docx]

**Supplementary material 1.** Univariate analyses of prognostic factor for DFS and OS in women with apparent early-stage clear cell carcinoma of the endometrium.

|  |  | DFS^1^ |  |  |  | OS^2^ |  |
| --- | --- | --- | --- | --- | --- | --- | --- |
|  | HR^3^ | 95% CI^4^ | *P* |  | HR | 95% CI | *P* |
| Age at diagnosis |  |  |  |  |  |  |  |
| < 65 years | Reference |  |  |  | Reference |  |  |
| > 65 years | 1.49 | 1.03-2.16 | 0.037 |  | 1.72 | 1.21-2.81 | 0.004 |
| Marital status |  |  |  |  |  |  |  |
| Married | Reference |  |  |  | Reference |  |  |
| Single | 1.83 | 1.25-2.67 | 0.002 |  | 1.459 | 0.96-2.22 | 0.078 |
| Unknown | 1.60 | 1.01-2.55 | 0.046 |  | 1.572 | 0.96-2.57 | 0.070 |
| ASA^5^ physical status score |  |  |  |  |  |  |  |
| I/II | Reference |  |  |  | Reference |  |  |
| III/IV | 2.34 | 1.68-3.27 | 0.000 |  | 2.65 | 1.83-3.85 | 0.000 |
| Stage (FIGO^6^ 2009) |  |  |  |  |  |  |  |
| I/II | Reference |  |  |  | Reference |  |  |
| III/IV | 3.87 | 2.70-5.53 | 0.000 |  | 4.43 | 2.94-6.67 | 0.000 |
| Tumor size |  |  |  |  |  |  |  |
| < 2cm | Reference |  |  |  | Reference |  |  |
| 2cm - 4cm | 1.62 | 1.11-2.38 | 0.013 |  | 1.71 | 1.11-2.63 | 0.014 |
| > 4cm | 3.07 | 1.99-4.74 | 0.000 |  | 3.64 | 2.28-5.80 | 0.000 |
| Peritoneal cytology |  |  |  |  |  |  |  |
| Negative | Reference |  |  |  | Reference |  |  |
| Positive | 1.05 | 0.62-1.77 | 0.851 |  | 1.14 | 0.63-2.07 | 0.667 |
| lymph-vascular space invasion |  |  |  |  |  |  |  |
| No | Reference |  |  |  | Reference |  |  |
| Yes | 1.13 | 0.73-1.77 | 0.583 |  | 1.46 | 0.85-2.51 | 0.275 |
| Nodal involvement |  |  |  |  |  |  |  |
| No | Reference |  |  |  | Reference |  |  |
| Yes | 2.22 | 1.58-3.14 | 0.000 |  | 2.26 | 1.55-3.30 | 0.000 |
| Surgical approach |  |  |  |  |  |  |  |
| Open | Reference |  |  |  | Reference |  |  |
| Laparoscopic surgery | 1.42 | 1.01-1.98 | 0.043 |  | 1.09 | 0.75-1.61 | 0.641 |
| Systematic lymphadenectomy |  |  |  |  |  |  |  |
| No | Reference |  |  |  | Reference |  |  |
| Yes | 0.54 | 0.38-0.76 | 0.000 |  | 0.58 | 0.39-0.85 | 0.005 |
| Omentectomy |  |  |  |  |  |  |  |
| No | Reference |  |  |  | Reference |  |  |
| Yes | 0.80 | 0.45-2.38 | 0.465 |  | 0.68 | 0.50-1.98 | 0.307 |
| Adjuvant therapy |  |  |  |  |  |  |  |
| No | Reference |  |  |  | Reference |  |  |
| Chemotherapy | 0.58 | 0.38-0.90 | 0.014 |  | 0.54 | 0.35-0.89 | 0.021 |
| Radiotherapy | 0.68 | 0.36-0.89 | 0.003 |  | 0.63 | 0.35-0.92 | 0.037 |
| Chemoradiotherapy | 0.42 | 0.22-0.79 | 0.008 |  | 0.39 | 0.18-0.82 | 0.013 |

1 Disease-free Survival

2 Overall Survival

3 Hazard Ratio

4 Confidence Interval

5 American Society of Anaesthesiologists

6 The International Federation of Gynecology and Obstetrics
